# How to Report Methods to an Interdisciplinary Audience: Machine Learning

**DOI:** 10.1016/j.isci.2020.100909

**Published:** 2020-02-20

**Authors:** Simona Fiorani, Stefano Tonzani

**Affiliations:** Lead Editors, *iScience* by Cell Press

With the explosion of artificial intelligence, machine learning techniques have become ubiquitous, in disciplines ranging from materials science to oncology. However, sometimes the methods sections of papers stemming from application of machine learning algorithms are hard to understand, and it is often difficult to appreciate “at a glance” whether the work has been performed to a high standard.

Since iScience is an interdisciplinary journal, our editors and Editorial Board see articles from disparate disciplines as a matter of course, and we have developed ways to make sense of this variety by asking ourselves (as well as our authors and referees) certain questions. This Editorial is the first one of a series of write-ups about what our Editors look for when we scan papers reporting on common methodologies that are used in many different areas of science. The intent of the series is to increase transparency of reporting, facilitate Editors' and Reviewers' assessment, and reduce overall the time to publication, since some of these items are typically pointed out in peer review. Our Editorial Board members (in this case Profs. Cenk Sahinalp and Shyue Ping Ong) are typically instrumental in informing these guidelines. We would also like to acknowledge conversations with Helena Deus at Elsevier.

When preparing a manuscript describing a machine learning approach, we suggest adopting comprehensive reporting guidelines. For biomedical research, an example is reported in J Med Internet Res. 2016 Dec 16;18(12):e323. For physical sciences research, we suggest looking into more general topical reviews offering critical appraisal of machine learning methods. There are also checklists to improve on machine learning reporting in the literature, as well as machine learning testing techniques and workflows. On the algorithmic side, the guidelines for the NIPS conference (one of the premiere venues to present machine learning research) are also useful in terms of characterizing novelty of the approach and quality of the insights.

In any machine learning application, the underlying dataset is key; thus, it is important to accurately describe the dataset used (including any pre-processing) and the split between training/validation/test sets. The training set should be balanced and generalizable. The computational model should be described in detail, and comparisons with the state of the art should be supplied.

We strongly encourage data and model/software sharing as part of the submission, either as Supplemental Information or uploaded to a suitable database (e.g., github). We also encourage formal citation of the datasets used, especially if they are stored in an external repository.

As Editors, we ask ourselves a series of questions, when looking into a paper using machine learning. The first, and most obvious: is this a problem that should be solved through machine learning? If yes, have authors established and articulated a rationale for the particular method they use? For each model used, how often can the authors “afford to be wrong” and how wrong can they afford to be? Do the authors reliably provide justification for the scoring metrics they used?

With respect to the methods, it would be very difficult to have comprehensive guidelines that encompass all uses of machine learning, because these can be as diverse as classification/clustering (assigning a category to each data instance), regression (predicting a value for each data instance), dimension reduction (reducing training complexity), and control (controlling actions to maximize rewards). However, there are a set of items we look for in the methods:

1. Is the paper dealing with categorical or quantitative variables? What is the computational model used? Is it applicable to the dataset? If convolutional neural networks are used, how many layers are employed?
2. Is regularization used? What type? Have the authors initialized the model at different points?
3. Are data sources clearly defined?
  a. Prepared in a way that is transparent and has a valid rationale
  b. What data cleaning (if any) was applied?
4. Are training, validation, and test sets separate?
5. What is the size of dataset? Is there a comparison between data used and ground truth?
6. Training data: is it
  a. Balanced
  b. In distribution
  c. Generalizable
  d. Looking far enough back
  e. Preprocessed?
7. Metrics for categorical variables: accuracy, precision, recall; is the “confusion matrix” (true-positive rate versus false-positive rate) present? Metrics for quantitative variables: cost function.
8. For multi-objective problems: are the tasks independent? If so, is the scoring method used reported?
9. Comparisons with state of the art.

The purpose of these guidelines is to increase the transparency of the editorial process and to give clear expectations for reporting. When the methods in a manuscript are not reported in a transparent manner, it is our duty as Editors to discuss with authors possible ways to improve, before the peer review stage.

As mentioned at the outset, the present Editorial will be the first one in a series about editorial guidelines for methods reporting. Many of the methods we will discuss are new or relatively new; therefore, the standards might not be fully agreed in the community of practitioners. We are keen to engage with the community to refine those guidelines and to help uphold good standards of reporting in interdisciplinary settings.

